# Notes on Belgian Lunatic Asylums, Including the Insane Colony of Gheel

**Published:** 1857-01-01

**Authors:** John Weester

**Affiliations:** Fellow of the Royal College of Physicians, a Governor of Bethlem Hospital, &c.


### Art. IV.—NOTES ON BELGIAN LUNATIC ASYLUMS, INCLUDING THE INSANE COLONY OF GHEEL.

By John Webster, M.D., F.R.S.,

Fellow of the Royal College of Physicians, a Governor of Bethlem Hospital, &c.

### Preface.

Having communicated in former numbers of the *Psychological Journal* various observations collected during several recent visits made to French and Scottish Lunatic Asylums, I am induced, by the notice they then received, again to resume a traveller's pen, in order to describe cursorily the present condition of different Belgian institutions inspected during last autumn; including particularly, the insane colony of Gheel, which seems not only the most remarkable, but is actually the oldest existing establishment for maniacs throughout Europe. Previous however to entering upon such tasks, one or two general remarks respecting the lunacy laws of the country just named, it is hoped may not prove inappropriate, or be thought altogether superfluous.

Like England and France, considerable alterations have been made, of late years, in the laws affecting lunatics, as also regarding the administration of Asylums in Belgium. The most important measure recently passed being the Legislative Act, dated 18th June, 1850; according to which, questions connected with lunacy institutions, and the management of patients attacked by mental disease, are now regulated. That enactment was followed, on the 1st of May, 1851, by an ordonnance of King Leopold, containing general and organic rules to explain more fully the previous law, and its practical application. In order to give British readers some idea of these new regulations, it may be briefly stated that, no person can either open, or at present direct, any institution in Belgium destined for the treatment of lunatics, without first obtaining the authorization of Government; whilst a similar permission must also be procured, for the continuance of all hitherto existing insane establishments.

Every house in which, not only several, but even one lunatic resides—not being a relative of the individual under whose care such person is placed, or unless with the curator, tutor, or provisional administrator—is considered an insane institution. Before granting a licence to receive lunatic patients into any building, it is required—First, that the situation be salubrious, the locality well aired, of sufficient extent, and have convenient interior arrangements. Second, that the sexes are separately lodged, and classified according to the nature or requirements of their respective mental maladies. Third, that medical and sanatory officers be attached to each establishment, with sufficient accommodation for the wants and condition of the inmates; and, Fourth, that the permanent deputation, appointed for such purposes, must renew, every three years, their approval of every professional attendant. This body, or "College," as it is now denominated, may also modify the medical staff, order the dismissal of any official, in cases of grave negligence; or should he have omitted to perform duties legally required. An appeal may, however, be made to the King against such sentence.

According to other regulations also in force, no insane person can be at present received into an asylum, excepting as follows: —First, upon the written petition of the tutor of an interdicted party, accompanied by a resolution passed in the "Conseil de Famille" for that purpose; but in cases where interdiction has not yet been pronounced, the provisional administrator must petition. Second, on the demand of a local authority empowered to grant domiciliary assistance to pauper lunatics. Third, in virtue of the decision by a local judge, according to the 95th article of the communal law Fourth, in execution of an order at the suit of a public ministerial officer. Fifth, upon petition of any person interested in the patient; but specifying the nature of their intimacy, the particulars of the alleged case, and any degree of relationship, or affinity, which exists betwixt the applicant and lunatic. All these documents being further countersigned by the commune Burgomaster, where such insane persons are found, or reside. Lastly, upon a decision of the permanent deputation appointed by the Provincial Council. In urgent cases, however, this order may, nevertheless, be made by the Governor alone; but, in all such examples, every fact must be reported to the Council, at their meeting next ensuing.

In each of the preceding modes, excepting that first named, a medical certificate must be likewise produced, describing the party's mental condition, whom it is proposed to place under treatment, as also every particular symptom characterizing the actual disease. This document should neither bear an older date than fifteen days, nor be signed by any medical officer of the establishment, to which the patient is consigned. However, in very urgent cases, the usual medical certificate may be dispensed with at the moment of admission; but its delivery is imperatively required within twenty-four hours subsequently.

Not later than one day after the patient's reception into an asylum, the chief officer must notify every such admission— 1. To the provincial governor. 2. The King's procureur of the arrondissement. 3. The cantonal judge of the peace. 4. The burgomaster of the commune; and, 5. To the committee of inspection. Besides these necessary formalities, the same official authority should intimate to the Procureur of the arrondissement, where the patient previously resided, the individual's admission; so that, through the local officers, he may inform the relatives or friends of that circumstance. A similar procedure being also adopted in cases of sequestration.

No lunatic can be sequestrated in his own domicile, neither in the house of a relative, nor of any person who may occupy that responsible position, unless the mental condition of such insane patient be certified by two medical practitioners, legally qualified. One being appointed by the party's family, or the persons most interested, the other by a cantonal judge of the peace, who must satisfy himself, through personal examination, respecting the lunatic's actual condition. Besides which, he is required to renew his visits at least every three months. Independent of these periodical inspections, that magistrate ought further to receive, every quarter, a fresh certificate from the family physician, reporting the patient's state, during the whole term of sequestration. Moreover, he may also order at any time another practitioner to visit the lunatic, whenever that proceeding seems necessary, or is considered expedient.

Every lunatic establishment, or temporary asylum, is visited by official persons specially delegated by Government for that purpose; or by the permanent committees of inspection appointed to carry into effect the recent regulations respecting lunacy. Each asylum, and the patients they contain, must be inspected at least once, as follows:—1. Every half-year by the Burgomaster of the commune. 2. Every three months by the Procureur of the arrondissement; and 3. Annually by the provincial Governor, or by one member of the permanent deputation of the provincial council, named by the governor. Temporary asylums, or provincial depôts for lunatics, are also inspected once every three months, by the Burgomaster of the commune in which it is situated, and by the cantonal judge of the peace, besides the official persons previously specified.

By existing regulations, when the aggregate inmates of each sex do not exceed fifty, there need be only two divisions of patients—viz., tranquil and agitated lunatics. Should the number of the same sex amount to more than fifty, there must then be four sections at least—namely, tranquil; agitated, and furious; idiots, including dirty patients; and, fourth, convalescents.

Whenever either sex comprise more than one hundred patients, then all clean and tranquil inmates must be placed separately from the dirty; whilst the idiots should constitute two distinct categories. For every ten patients, one seclusion cell is deemed indispensable, unless under particular circumstances; whilst private patients ought always to be kept apart from pauper inmates. Lastly, neither class must ever be mixed with boarders, not insane.

Each asylum should have a medical officer; and where two or more are attached, one is denominated the chief physician. If the house contains beyond one hundred patients, there ought to be an assistant physician, or one medical pupil, resident within the institution, or in its vicinity; by either of whom, all the patients should be visited daily. The medical staff, their emoluments, and attributes, are submitted every three years, during the month of November, to the permanent committee of the provincial council for consideration and approval; who may also make whatever changes they deem requisite. When the director of an asylum, whether public or private, undertakes to support the inmates, as also to superintend such an establishment, he must first obtain a special authority from the permanent deputation. Again, for every ten patients, there ought to be one attendant. Lastly, no person can erect a new asylum without the sanction of Government, or even make important changes in an old establishment, unless under a similar authority; and then only after plans of the proposed alterations have been forwarded for examination and approval.

With reference to medical certificates required, prior to the admission of patients into an asylum, it is expressly enacted that the parties signing such documents shall specify when the disease commenced, its nature, duration, and essential characteristic symptoms; whether the patient had undergone any previous treatment, and, generally, every other circumstance with which the medical officer should be made cognizant. In addition to this report, the certifying practitioner is expected to forward a sealed communication indicating the cause, known or presumed, of the inmate's malady, and if any relative has ever been affected with mental disease. When these certificates refer to indigent persons, they must be granted gratuitously, by the medical officers of the poor belonging to the particular locality, where such insane patients may reside, even casually.

Numerous other points, besides those now mentioned, are also specifically alluded to, in the legislative enactments just quoted; but, being of secondary importance, the subject need not be pursued further. My object, at present, being only to speak generally of the system which prevails in Belgium with reference to lunatics, and the organization of asylums, not certainly to tire readers by any lengthened legal disquisition; believing that proceeding would seem both misplaced and supererogatory to the chief purpose proposed for discussion in this communication.

Nevertheless, before adverting particularly to the institutions recently visited, it is important as a preliminary to remark that the special inspection of every lunatic asylum—whether provisional or permanent, as also temporary depôts for the insane, during their transference from one district to another—is confided in each arrondissement to a committee, consisting of five, seven, or nine members, including the district Commissary, who sits officially. All are nominated by the King, while the half is renewed every two years; but retiring members may be reappointed immediately. These Committees select their own secretary, the Commissary being always chairman, and having a casting vote in cases of equality. That officer alone summons the committee, names the hour, their place of meeting, and, in case he cannot attend himself, appoints a substitute to preside. In short, the above government official is the moving power of this local board of inspection. It corresponds directly with the Minister of Justice at Brussels; and the united committee is required to visit, at least once yearly, every lunatic asylum situated within their own particular jurisdiction. Besides these annual visits of the entire local committee, individual members must arrange a rota amongst themselves, so that each insane establishment shall be officially inspected, not seldomer than every two months. Such inspections must not be mere formalities, as they embrace many important questions of inquiry and examination, which are specifically enumerated in the new code of lunacy regulations already quoted.

Finally, but independent of these local committees of examination, and in conformity with the Legislative Act, dated 18th June, 1850, a general commission has been instituted, whose duty is to inspect every asylum in Belgium, and report respecting their actual condition. These Commissioners are appointed by royal decree, and receive instructions from the Minister of Justice, to whom each are responsible; their salaries and all expenses incurred being included in the budget of that department. The present commission comprises three members—viz., M. Ed. Ducpetiaux, also Inspector of prisons; Dr. Joseph Guislain, well known throughout Europe for his great reputation; and M. D. Sauveur, also medical Inspector at the Home Office; with M. N. Oudant, as secretary. In addition to visitng officially asylums, and taking special cognizance of everything connected with lunacy, it is also one of the chief functions of these gentlemen to present, annually, a detailed statement regarding the insane establishments placed under their surveillance, which is first communicated to the Legislative Chambers by the Minister of Justice, and afterwards printed. The last Report issued is that for 1855, which amply merits perusal, as an able and instructive public document.

According to that ministerial paper, the total number of receptacles for lunatics, whether public or private, amounted to fifty-one last December. Of these, seventeen were appropriated to patients of both sexes; fifteen to male, and nineteen to female inmates, exclusively. Again, thirty-two of the above admitted both pauper and private patients; five only indigents; and fourteen received none but members of the middle, or those belonging to the upper classes of society.

Many of the above enumerated institutions are of limited extent: since only eight contain from 100 to 150 inmates; whilst the population of not more than six actually exceed 200 patients. The largest establishment at present is St. Julien, in Bruges—if the insane colony of Gheel be excluded; which latter locality cannot be classified comparatively,—seeing, the lunatics congregated in that district are distributed both in numerous private houses of the town, and amongst neighbouring villages, as I shall more particularly notice in the subsequent part of this communication.

Speaking generally, Belgium does not possess asylums placed upon the same footing as in France, and in many other European countries—where the direction, and also the management, are confided to agents appointed and paid by Government. In this part of the Continent, the institutions for lunatics, at present open, belong either to hospitals, private individuals, or religious associations; who administer them at their own risk and expense. These peculiar circumstances have created difficulties in carrying out some of the Commissioners' late recommendations, made with a view to improvements; and may in part explain why several old institutions still remain nearly in the same "*deplorable condition*" they exhibited, when the Commissioners, about three years ago, made their first official inspection, after being appointed by Government.

Throughout Belgium, whose present population verges on 4,520,000 inhabitants, there were very recently 4907 recognised lunatics,—which hence gives a ratio of one insane patient to every 920 persons; the proportion being, however, greatest in urban, and least in rural communes. The latest statistical calculation made, with reference to this point, shows that in most towns, the amount reached to one lunatic for every 470 residents; whereas, only one insane person was found amongst so many as 1368 inhabitants of rural districts. In regard to sex, the males preponderated; their number being 2630—whilst only 2277 were females: thus giving a difference of 15·50 per cent., or nearly one-seventh more male than female lunatics. This fact is interesting, since it proves, notwithstanding the greater tendency considered to prevail, in most countries, of females to mental diseases, in

Belgium, generally, the predisposition seems strongest amongst the male part of its population. Of the 4,907 lunatics above enumerated, 1,220 were classed as private patients, or pensioners; the rest being indigents. About one-third were deemed curable cases; the remainder comprised incurables.

By way of giving an outline of the general movement which characterized the insane population under treatment, during one year, in all the asylums of Belgium, that of 1854 may be quoted as instructive. During the period above named, 1309 new patients are reported to have been then admitted, of whom 402 were discharged cured, being exactly 30 per cent. on the admissions; whilst 421 died; which, therefore, makes the mortality amount to more than 32 per cent., and greater than the actual recoveries, when similarly calculated. This result cannot be reckoned as by any means satisfactory, and it also becomes certainly difficult of explanation. Amongst the 1309 new patients received into the different establishments, 1142 were cases who had never been previously insane, whilst 167 were reported relapses; thus making nearly one-eighth of the entire number.

Although impartial observers acknowledge that numerous ameliorations still require to be accomplished, in various Belgian institutions for the insane,—both private and public,—it is not the less true, many important and useful improvements have been effected since the permanent commission of inspection was first appointed. These public authorities state in their last Report, amongst other facts which are gratifying, that "Thanks to the changes made in the insane establishment of the Cellite Frères at Antwerp; the hospice at Duffel; the asylum for males in Louvain; that of Menin; St. Nicolas, in East Flanders; Strop, near Ghent; as also the institution of Uccle and Evere, in the environs of Brussels, the number of inmates have increased in a proportion more or less remarkable. On the contrary, those receptacles for lunatics which do not yet supply all desirable guarantees of their good condition, will be inevitably abandoned." The parties here alluded to must soon comprehend their real interest; so that, in accordance with the dictates of humanity, beside future material prosperity, they will be compelled to introduce those reforms the new laws prescribe, and which are likewise imperatively required for the physical comfort of many afflicted inmates, now confined in several unlicensed establishments.

Unlike most other European capitals, Brussels possesses no public asylum for the permanent reception of lunatics; there being only a small provisional depôt attached to the civil hospital of St. John, which is more like a prison than an insane receptacle, and where mad patients are temporarily confined, previous to their transference to other establishments; generally to Gheel or

Bruges. When I visited this temporary domicile, it contained only ten inmates; some of whom had been merely placed within its precincts, prior to removal elsewhere. Indeed, only a few days afterwards, I recognised two of these identical patients at the provisional infirmary of Gheel, where they had been sent, preparatory to being placed with some authorized resident in that commune.

Indubitably, ample accommodation may be found, for insane members of the middle and upper classes, in the private "maisons de santé," near Brussels. For instance, in that of M. Denaeyer-Dupont, at Evere, containing, on an average, fifty-six inmates; or at the larger institution belonging to M. Vanderkindere, having upwards of eighty patients, which occupies an elevated, salubrious position, not far from the capital. Here, the general aspect, means of treating insane patients, and also various modern appliances, seemed very satisfactory when I visited the establishment. Again, should parties feel desirous of sending their relatives to new scenes or more distant localities, then, the excellent "maison de santé," at Ans et Glain, near Liège, the property of M. Abry, and whose son-in-law is the resident physician, may be selected. The latter institution now specified occupies an admirable position, possesses an extensive yet beautiful prospect over the neighbouring city, as also the fertile valley of the Meuse; and having personally examined this precinct and buildings—then containing sixty-five patients—I can speak favourably of its several capabilities. Notwithstanding such varied means for treating demented persons—not victims of poverty in addition to their mental diseases—still, the absolute want of a large public asylum for indigent lunatics, in such a populous locality as the metropolitan district, is remarkable; particularly when readers remember that in the arrondissement of Brussels, containing a population of about 415,000, there are nearly 600 insane persons reputed natives, most of whom now occupy asylums in other provinces. So great a desideratum requires some speedy remedy, for the sake of humanity, altogether irrespective of other equally potent considerations.

Although the superior Administrative Board of existing Brussels Charitable Institutions have not yet come to any determination respecting this deficient accommodation for lunatics within their own jurisdiction, that question has not been overlooked. Indeed, various members of Council, the Inspectors of lunatics, and also the Provincial Governor, it is said, seem fully impressed with the great importance of constructing an asylum of the first rank, for receiving indigent patients, which shall in future obviate any necessity of sending their insane poor elsewhere. The case is urgent; and however great might be the preliminary expense which such an establishment must entail upon the Brussels hospital administration, it ultimately would prove most beneficial, and relieve the city from all opprobrium of being now obliged to solicit admission for their necessitous insane patients into the asylums of other districts. This deficiency ought to be supplied, whereby the metropolitan province of Brabant shall no longer remain without having a public asylum, supplying adequate accommodation for the insane poor born on its soil; and who, therefore, possess the strongest claims to participate in the benefits which such an establishment would disseminate.

Considering the limited extent of Belgium, the aggregate asylums for the insane it contains are much more numerous than in almost any other European country. The institutions are, however, generally of small size; nay, many have only from ten to thirty inmates. The largest numbers are located in East and West Flanders; the chief places being Ghent and Bruges, or in the immediate vicinity of these towns. Of course, this remark does not apply to the insane colony of Gheel, which is situated in the eastern part of the province of Antwerp, not far from its frontier towards the Rhine, and contains more lunatic patients than any other district; but the inmates are there very differently placed, being lodged with cottagers, peasants, and others—not congregated together in a confined public asylum.

The above facts, and recent investigations respecting the number of lunatics under treatment in different insane establishments, besides those which still remain with relatives, prove that mental diseases are by no means of unfrequent occurrence throughout Belgium; and, if compared with neighbouring kingdoms, they appear even more numerous. The ratio, as already stated, amounts to one lunatic in every 920 inhabitants; which, therefore, constitutes a higher proportion than in France, Germany, or England. The causes of this marked frequency of insanity amongst Belgians, not being one of the objects proposed in these notes, I consequently only allude to the question, from considering it of much interest, and deserving farther discussion. Nevertheless, hereditary tendency to mental disease, the prevalence of scrofula amongst the lower classes, their poor innutritious diet, frequently more vegetable than animal, weakened physical frames—too often caused by hard work, and privations in the labouring population—with the mixed or mongrel races which seem to characterize many natives of this country, must exert considerable influence, unquestionably. These peculiar features certainly attracted my special observation, when recently travelling through Belgium. In its large prisons—many of which were inspected, in mendicity, or poor-houses, lunatic asylums, the churches—where crowded congregations then often assembled,—

at railway stations, and in market-places this occurred. Indeed, wherever numerous bodies of spectators got collected together, even casual observers could not avoid noticing the diversity of race, and outward physical aspect, which the populace around then supplied for ethnological meditation. The dark hair and swarthy features of Spain; the blue eyes, light auburn locks, and true Saxon countenances; the complexion, gait, and manner of genuine natives of France; and, lastly, the more staid, phlegmatic mental and bodily characteristics of Dutchmen, might be everywhere easily distinguished. In short, throughout no country of Europe, which I have ever visited, was the same difference of peoples so peculiarly observable, as seemed to prevail in the places under discussion.

Before describing the several public asylums which form the subject of subsequent remarks, it may be premised, with reference generally to Belgian establishments for the insane that, amongst the fifty-one asylums now open, three-fourths are situated in towns, or their immediate environs; while only about one-fourth occupy rural communes. From this cause, their precincts are often of very limited extent; and, consequently, such institutions become badly adapted for the treatment of lunatics. This remark particularly applies to Ghent, and likewise to Bruges; although to the latter city, less strongly. However, as in these districts the largest public asylums are situated, they therefore will form the chief subject of future observations. To notice small establishments, which contain very few inmates, would prove superfluous; consequently, I will at once proceed to describe the two rather extensive institutions for lunatics, located in the ancient and once powerful capital of West Flanders—namely, Bruges.

Preliminary, however, to commencing that undertaking, it seems desirable to give some outline of the features which most Belgian asylums exhibited very recently, in order to contrast their former state with the present. No authority in reference to such matters can be considered so truly unexceptionable, and less liable to express exaggerated or unjust condemnation of the public asylums in Belgium, than a native of that kingdom; since his feelings would be naturally inclined to take an opposite direction. A more trustworthy and also impartial judge cannot therefore be found, or one better able to speak upon the subject with weight, than M. Guislain, who says, in his first lecture, "Sur les Phrénopathies," published 1852, "Lunatics in Belgium remain forgotten in sombre prisons. They resemble merchandize amongst speculators, who make them an object of infamous traffic, like animals from the farm-yard, fit only to be bought and sold, as horses or swine. Much talk has certainly taken place during the last thirty years; but so little has yet been accomplished that our afflicted maniacs have been only turned round in a vicious circle of selfish and fatal administrative influences." To show that asylums are now improved, I commence with those at

### Bruges.

In this formerly opulent city, and, several centuries ago, a great emporium of trade, with upwards of 150,000 inhabitants—but now reduced to less than one-third of that number, whilst its commerce is almost annihilated—there are at present two extensive institutions for treating lunatics—namely, St. Julien, and St. Dominick. Having visited both asylums early last September, I therefore propose giving a brief account of the inspection then undertaken.

1. *St. Julien Asylum.*—This institution is one of the most ancient establishments for receiving lunatics, throughout Belgium. It is situated in a wide, airy street, near the railway station, close to the Porta Santa—one of the gates of Bruges—and closely adjoining its ramparts. Being originally a convent, the buildings are old, and some appeared not well adapted for their present purpose. Still, considerable improvements in the interior arrangements having been since effected, it is much resorted to by patients of both sexes. According to tradition, this locality formed a hostelry for pilgrims, so early as the seventh century; but it was not till about A.D. 1500, that insane persons were received within its precincts for protection and treatment. Attached to the present lunatic institution of St. Julien, and under the same superintendence, two other—although much smaller—establishments, are also opened for the treatment of persons afflicted with mental disease. One is that of St. Anne, situated in a healthy and agreeable district near Courtray; the other being the Convent of Cortenbergh, lying between Brussels and Louvain, in a very picturesque locality, celebrated for salubrity. This house has been recently rebuilt, according to the approved principles of modern architecture; but, being intended solely for the accommodation of female patients of the upper and middle classes, the number received is therefore very limited. Having thus three separate establishments—all under the same superior direction—the relatives of private patients may therefore secure, if considered advisable, a change of residence, so that those who wish can then pass the winter in town, and summer in the country.

When I visited St. Julien—early last September, the total population of the chief institution, situated in Bruges, amounted to 310 lunatics; of whom 166 were male and 144 female inmates. Of these, half were tranquil patients, seventy-five agitated, thirty-eight epileptics, thirty idiots, and twelve were then considered convalescents. Amongst the whole, thirty were classified as dirty persons; the sexes being nearly equal, in reference to that particular feature. No female lunatic appeared in camisole, or undergoing any kind of bodily restraint whatever. However, one male patient was temporarily confined by a strait-waistcoat, whilst two men and one woman were in seclusion cells; all three being much agitated and very violent. The general population seemed tranquil, considering the number of inmates congregated in different divisions. Many females occupied themselves in lace-making, domestic employments, and in preparing or mending clothes for residents. A large number of male patients were engaged in agricultural work on the adjoining farm, which amounts to twenty acres, belonging to this institution; as likewise in the garden attached to the building for private male pensioners. These pay a larger sum for board than the indigent residents, and varies from 500 to 2500 francs annually; whereas, the allowance received from communes, for pauper patients, amounts to only 75 centimes per diem—that is, 273 francs, or 11*l.* annually; which truly seems a very low remuneration for such inmates—feeding, lodging, and clothing included.

Being in most parts an ancient structure, this asylum is not conveniently arranged. The apartments are too crowded in several instances, and its buildings being sometimes very close together, there seemed not sufficient separation of several wards occupied by the different sexes. Nevertheless, much has been done to remedy existing defects; and considerable improvements are also in contemplation. The patients' court-yards are four in number, some being, however, rather limited; and there are, besides, three small gardens for inmates taking open-air exercise, with another of greater magnitude for pensioners, whose number amounted to forty-eight, comprising twenty-two females, and twenty-six male lunatics. Of these, several were, I understood, natives of Great Britain. Indeed, one was pointed out who had only recently arrived from the north of England.

Two physicians and one surgeon are attached to the St Julien Asylum, one of whom pays daily visits, or oftener, if necessary; but there is no resident medical officer. The chief authority and director is M. le Canon Maes, who has a lease of his present premises from the Mendicity Depôt of Bruges. That reverend gentleman may be therefore considered the proprietor. He is principal manager, takes all pecuniary risk upon himself, and must be at whatever expenses either improvements or alterations may entail. Those now essential are certainly considerable, in order to meet the requirements of constituted public authorities; and, consequently, to render the interior more in unison with the present ideas entertained, regarding what seems proper treatment for lunatics.

Having been only provisionally licensed until the 1st of last April, on condition that various important changes, admitted by impartial parties as urgently required, were effected in its internal arrangements, this institution remains at present without legal sanction ; and will continue, till the Committee of Inspection's suggestions are completed. Different propositions were made to arrive at a satisfactory solution, but, hitherto, every effort has proved unsuccessful. As the Communal Council of Bruges have not yet sanctioned any of the plans proposed, and as the administrators of hospital property, the Inspectors of lunatics, besides the parties interested pecuniarily in this establishment, all entertain very different opinions with reference to the questions in dispute, some time may yet elapse ere matters shall be arranged satisfactorily. This dilemma is much to be regretted, since the hospital of St. Julien has long been known as a useful institution ; and if properly reorganized, whilst various admitted defects were removed, it would doubtless confer most useful benefits upon those unfortunate persons, for whose individual advantage it is destined. The anomalous position, in which this institution is now placed, forms the subject of a special notice in the Committee of Inspection's last Report, who think it cannot much longer exist as at present. The ameliorations demanded must be carried out efficiently, or the establishment will be shut up and suppressed.

During the past year fifty-two new patients were admitted, thirty-two being male, and twenty female lunatics; twenty-seven left the asylum cured, of whom nine were male and eighteen female inmates, and thirty-three died ; the male patients in that category being twenty-one in number, with only twelve females. These figures hence show that insanity oftener affected male persons applying for relief at this institution, and fewer were discharged cured; whilst the proportion of deaths ranged higher amongst that sex, than those recorded in female patients. Such results, however, become less remarkable when it is known that two-thirds of the inmates were classed as incurable lunatics ; and in about one-third only was a slight hope entertained of ever doing much good, still less gave any prospect of recovery. In fact, the mental diseases of many being of long standing, their favourable termination consequently appeared utterly hopeless.

2. *St. Dominick Asylum.*—This institution—like the former, also an ancient convent—is situated in one of the streets of Bruges, and has been now appropriated for the treatment of insane patients upwards of half a century. Since 1846, the asylum has received considerable augmentations, in reference to accommodation; and, at the same time, various ameliorations have been effected in its interior arrangements. Nevertheless, from the buildings being defective—some of which appeared rather ancient—and although several new constructions have been recently erected, this establishment is not considered well adapted as a residence for private patients. Hence, the proprietors, who are five in number, have lately leased a château named "St. Michel," with a garden and farm of about 100 acres attached. This "maison de santé" is nearly two miles from Bruges, on the Courtray road, and had, when I visited it, twenty-nine male pensioners, as also twenty convalescent patients of the indigent class, sent from the town establishment to labour in the fields; which work often materially promotes their ultimate recovery. The central asylum likewise receives, according to an arrangement with the Department of Justice, lunatics accused of crimes, and those who have been convicted by ordinary courts of law, or sent from various prisons. This criminal category forms a separate section, and quite distinct from other inmates; whilst such parties are placed in courts or cells specially constructed, to prevent escape.

When I inspected St. Dominick, the population comprised 330 persons, consisting of 182 male, and 148 female lunatics; amongst the latter sex eighteen being pensioner patients, belonging to the upper and middle classes. Besides these numbers, twenty-nine insane men, paying from 500 up to 3000 francs annually, with twenty indigent lunatics, occupied in agricultural labour, as previously stated, were then lodged at St. Michel's; so that the total inmates of the united establishments under discussion, amounted to 379 individuals. In the town department, the patients are divided into five categories; viz., 1st, convalescent; 2nd, tranquil lunatics; 3rd, agitated; 4th, turbulent; and 5th, idiots, with dirty inmates. The same classification being adopted in both sexes throughout.

Again, in reference to the nature of their mental maladies, according to information supplied to my inquiries, it appears twenty-one were epileptics, ten being males and ten females; twenty-eight men and twenty-six women were classed as dirty patients; thirty males and twenty-eight females as agitated; whilst only two male and one female inmates were said to be paralytic. The remainder being all reported tranquil lunatics; a large proportion of whom consisted, as elsewhere, of chronic cases, and considered incurable. The buildings comprised twelve court-yards, six being appropriated for male, and six for female residents; the agitated, and those requiring more surveillance than the rest, occupying very properly the central portion.

Some dormitories contained forty beds, others only fifteen, but all appeared clean, and also comfortable: particularly, when it is remembered the inmates were chiefly of the pauper class. The sleeping chambers for dirty patients were uniformly single-bedded, well ventilated, entirely free from any offensive odour, and seemed really much better than I have occasionally observed in other countries, for that class of lunatics. The general aspect of the asylum appeared most satisfactory : both male and female residents being also neatly and properly clothed; whilst the physical health of all was reported particularly good. No female amongst the entire population being sick or in bed ; and only one male invalid, slightly indisposed from bodily disease, occupied the infirmary, along with a soldier, almost convalescent from an attack of intermittent fever he had caught when in garrison at Newport ; where that malady proved, as usual, very prevalent during the recent summer, and of which he had become the victim, besides labouring under severe mental disease. Five female patients were confined in camisoles—but free, and walking about in the agitated court-yard ; another being in temporary seclusion. No male lunatic was in any way physically restrained, although two excited maniacs occupied seclusion cells, having become very excited and violent. However, this proceeding would not likely be of long continuance, and merely till they got more tranquil. If not entirely abolished, restraint is now as little employed as possible ; the general opinions respecting camisoles, and their utility, being much the same in this country as in France. Further, in the cases now mentioned, the strait-waiscoat was not tightly, but loosely put on : a great object being, apparently, to prevent the patient from injuring either others or themselves ; and chiefly to confine such parties' hands, so as thus to disable them from tearing their clothes, or so forth. Notwithstanding the number of agitated patients, the appearance of the entire population seemed that of quietude. The females everywhere were certainly more noisy and talkative, than the male inmates. But comparing this establishment with analogous collections of insane residents throughout France, there prevailed much less violence and excitement than I have often observed in that country, when visiting similar institutions.

Occupying and amusing the lunatics always constitute a principal object in the treatment pursued. Many male patients are consequently employed as tailors, weavers, spinners, and in other employments ; besides numbers also in the gardens, or at agricultural labour. One interesting and peculiar feature deserves however special notice—namely, the numerous former patients who have now become assistants, or " aides infirmiers," in the different wards : of whom, not less than thirty male lunatics of this description are so inscribed, according to a recent statement. Amongst female patients, the same system is pursued ; hence, not less than sixteen were also registered as assistants, on their own side of the institution.

Numbers were likewise engaged in the laundry, knitting stockings, making clothes, for other patients, as also in the kitchen; whilst all the bread consumed in this large establishment being made on the premises, the bakehouse therefore forms no inconsiderable means of employment to inmates. According to present opinions entertained by the executive authorities of this asylum, physical labour, as a means of distraction, exercises often most beneficial influences upon the mental condition of lunatics; consequently, it is always very zealously promoted. Nevertheless, no person is ever forced to labour, either through moral or physical restraint; that object being always attempted by the aid of example, or the desire of gain in those who are induced to work, and by granting small favours, with additional indulgences to the most industrious. Amusements and recreations are likewise assiduously promoted. Card-playing, draughts, dominoes, billiards, and gymnastics being very often resorted to as favourite sources of enjoyment.

The medical staff of this asylum consists of a chief physician, Dr. Van Hecke—well known as an experienced practitioner, resident in Bruges,—with two assistant physicians, and one consulting surgeon; while the lay officials comprise a director and secretary, besides an almoner. By way of conveying some definite idea of the number of persons employed, and hence actually required in managing such an extensive establishment as that of St. Dominick—containing always upwards of 300 lunatic inmates—it may be interesting to mention that, on the male side, besides the chief overseer, there are constantly twelve attendants, of whom nine superintend the workshops and garden, with six assistants, and six sub-assistants; irrespective of various convalescent patients, who also give their services in different departments. On the female side, in addition to the lady-superior, who overlooks all the others, there are thirteen religious sisters of the order of St. Dominick. These superintend the different wards, one in each, as also the clothing department, the kitchen, the laundry, the work-rooms, and dining-hall. The above sisters have, besides four head domestics, an unlimited number of female servants, taken from convalescent patients, upon a similar plan to that pursued in the male department. Not being permitted, by superior authority, to have "*sœurs* and *frères religieux*" in the same institution, all the attendants on indigent male patients consist of laymen. However, at the succursal asylum of St. Michel, where only male inmates are admitted, six religious brothers, with four lay-domestics, placed under the superintendence of a clergyman, manage the establishment; whilst a physician—Dr. Beckman—living in the neighbourhood, takes charge of all medical treatment and professional surveillance.

In order to obtain well qualified lay-attendants, and in sufficient number, when convalescent patients exhibit an aptitude, or express any wish to become regular domestics in this asylum, rather than leave, such parties are first placed on the list of candidates; when they obtain a particular dress, assigned by way of distinction. After fully proving their fitness for office, and so soon as a vacancy occurs, they are then installed as effective attendants. The above system has hitherto answered admirably; most of the present male servants employed at St. Dominick having been formerly patients. It is hence specially mentioned as worthy of trial, and if approved, for adoption elsewhere; since nothing is confessedly more difficult than to obtain good attendants on the insane; whereas, the result here has proved quite otherwise.

One feature at this institution also deserves special mention,—namely, the excellent medical register at present kept of all cases admitted. Such proceeding, certainly, is only in accordance with the recent law; but as similar documents seemed not invariably forthcoming elsewhere, and, I fear, do not always even exist in the form required, more credit is therefore due to Dr. Van Hecke, for the manner in which these valuable memoranda are officially preserved. I looked over several, and found records of symptoms, and treatment; besides *post-mortem* reports, which were most interesting. This repertorium of facts is already large: and doubtless will every year become more valuable to the practical physician, as likewise to zealous psychological pathologists.

Although not of much apparent significance, nevertheless, as it shows the great attention paid, even to minute matters of detail, a very recent improvement, or rather an important addition, which has been made to the male wards of St. Dominick, deserves being specially mentioned. *Spittoons are now placed in such apartments*, particularly those occupied by dirty patients. In this country, where almost every man and boy, nay, even women, seem slaves to that degrading, filthy custom, and health-destroying—both of body and mind—abomination, *Tobacco-smoking*, these appendages become absolutely essential throughout any inhabited dwelling, whether for sane people or maniacs. Therefore, irrespective of sanatory considerations, as most lunatics, by thus placing such saliva recipients within easy reach, may be taught to use them, instead of soiling floors or walls with their offensive spittings, internal discipline thereby becomes materially promoted. I am no advocate of smoking; on the contrary, would strongly condemn such an unseemly habit—or vice, more correctly speaking—from believing it proves both injurious to the mental faculties, and inimical to the physical powers of many votaries. Nevertheless, if mankind will obstinately use this deleterious weed, assuredly the most excusable proselytes are lunatics.

Therefore, spittoons ought always to be placed in every similar institution where smoking is permitted.

During the past year, 100 new patients were admitted into both establishments; sixty-one being male, and thirty-nine female lunatics. The total cures amounted to forty-four cases, of whom thirty-three were male, and eleven female inmates; while thirty-six deaths were recorded, twenty-three being of male, and thirteen female residents. It thus appears that the ratio of recoveries was forty-four, and the deaths thirty-six per cent.; when both results be calculated, according to actual admissions. More fatal cases occurred during June, October, and February, than throughout any other months of last year; whereas, the fewest happened in May, August, and December. The total number of persons who passed through the infirmary in the course of twelve months, from being attacked by bodily disease, was seventy-nine, of whom thirty-six died, as already stated, and thirty-eight recovered; thereby leaving five patients inmates of that department on the 1st of January; thus showing that physical disease prevailed here much more frequently throughout the former, than during the present season.

Having stated in a previous paragraph that the strait-waistcoat and personal restraint are not yet entirely laid aside at the St. Dominick Asylum, it may be now mentioned as instructive, and also further to illustrate the above important question, bearing upon the treatment of lunatics, that in this institution, where usually about fourteen to every 100 inmates appear agitated patients, the cases are but rare for which the medical officer feels obliged to institute coercive measures; seeing, cellular isolation generally proves sufficient. When bodily restraint is actually used, the camisole, or leathern bracelet, are the only means employed; and then chiefly in suicidal persons, and excited erotomaniacs. With reference to the application of physical coercion, such as those just mentioned, it was reported by Dr. Van Hecke, that amongst 377 lunatics under treatment, during the entire year, eighteen male and twenty-four female patients were subjected to cellular repression; which, therefore, represents a totality of fifty-four days. Further, eight men and ten women were confined by strait-waistcoats; whilst twelve male and sixteen female lunatics temporarily wore leathern bracelets. Lastly, eleven patients had been put in camisole, during two to four days consecutively, besides seven others for a much longer period. These authentic and official reports, showing the actual employment of personal restraint at this asylum, would be considered excessive in England, or altogether unnecessary, if not reprehensible. Still, it should be remembered that, throughout various continental countries, the application of camisoles, in furious or dangerous maniacs, becomes not only justified by several conscientious and experienced practitioners, but then even strongly defended from being, according to their opinion, both beneficial in repressive results under the above circumstances, and likewise proves often humane in its judicious application.

### Ghent.

Another district where numerous lunatics are at present congregated, in different asylums, is the ancient city whose name has been given above. Within this populous locality—now designated the modern Manchester of Belgium—having upwards of 100,000 inhabitants, but formerly nearly double that amount, and deserving special notice by travellers on account of its historical reminiscences, valuable pictures, and venerable buildings, there are, besides two large public establishments for lunatics of each sex, the Hospice of "St. Jean de Dieu"—although actually of very limited extent; next, two small asylums attached to the great and little Béguinage; then, the "Maison de Santé" for females in Rue d'Assaut; and, lastly, that known as the "Strop," which is situated on a rising ground, not very far from one of the gates of Ghent, but where only male patients belonging to the middle and upper classes are received. These seven establishments generally contain about 630 insane residents, upon an average; the majority being female lunatics.

Before adverting to different institutions in Ghent, besides the fact that, a greater number of female compared with male lunatics are enumerated, it is important to add as an authentic observation that, mental diseases seem exceedingly common amongst its general population. Thus, M. Guislain says, there is one lunatic to every 302 inhabitants, which constitutes, therefore, an enormous proportion; indeed, much greater than in either Germany, France, or England, and altogether unique. Without attempting now to explain this remarkable circumstance, however singular it appears, I at once proceed to consider—

1. *The Asylum for Males.*—This public institution is situated almost in the centre of Ghent, having a sluggish canal on one side, and adjoining a broad street of considerable traffic on the other. It is quite close to the "Hôtel de Flandre," where I happened to take up my quarters. As the principal entrance can be only approached by a narrow lane, visitors may hence easily pass its antique gateway unobserved; which actually happened to myself, when first endeavouring to find the venerable-looking portal whereby I gained admittance. The building now appropriated for receiving male indigent lunatics was an ancient Alexien convent, constructed some centuries ago. This seems proved by its very old chapel, where the insane residents still assemble for divine worship, and which really deserves inspection by antiquarians, or any curious archæological amateur.

This entire property belongs to the city: and in everything appertaining to its administration, is represented by a Commission of the Civil Hospitals. Having been condemned by competent authorities, and likewise by public opinion, as wholly unfit for the reception of lunatics, any opinion in reference to many defects seems, therefore, supererogatory. However, I would only further remark, after quoting an observation of one of its own able officers, who says in a recent publication, "The whole structure offers an accumulation of arrangements the most deplorable," that whenever the truly magnificent institution for male patients, now erecting near the Bruges gate, in one of the city faubourgs, is completed, this antiquated building will be closed as a lunatic asylum, and appropriated for other purposes; one of which, report states, being a barrack for lodging the local fire brigade. But happen what may, the sooner every insane resident now confined within the precincts of this venerable convent gets removed to the new asylum, so much the better; as, then, all sombre recollections of this melancholy abode will at least have become matters of history, if they be not forgotten, by the present generation.

Being only kept open as an asylum until the new institution is ready to receive its present inmates, to make any remarks regarding the accommodation now supplied appears out of place and superfluous; therefore, without adverting to such questions, I would observe that, at the period of my visit to this receptacle, the total insane male lunatics amounted to 260; amongst whom 30 were epileptics, 21 agitated, and 16 dirty patients. All were indigents, excepting 45, who paid a moderate board; but even these did not, however, belong to the superior classes of society; every inmate of that category being now placed at the "Maison de Santé" of Strop, which is, although separate, still under the same management. The general health of residents was reported, on the whole, as satisfactory. Not more than twelve patients were sick in bed, whose physical diseases seemed of a mild description, and none suffered from any serious malady. One lunatic was in camisole, while another wore leather gloves, to prevent him tearing his own clothes. These were the only persons under bodily restraint; hence, speaking generally, the whole establishment exhibited a tranquil aspect, including the quarter appropriated to agitated and furious patients.

Respecting the causes of insanity in patients recently admitted, moral influences were reported the most frequent. Drunkenness being likewise often assigned. Dissipation, with misconduct, produced madness in several instances; and lastly, hereditary predisposition seemed to have existed in about one-third the total admissions. This transmissibility of mental diseases was, however, proved to prevail, in a greater ratio, from father to son, than from mother to her male offspring; thereby showing that, here as elsewhere, insanity oftener descended through the same sex than the opposite. Indeed, it was confidently said, grandfathers more likely transmit mental complaints than the grandmother to descendants.

In one of the court-yards visited, about twenty idiot boys were assembled, who seemed, on our entering, going through military evolutions, under the directions of a fugle-man. This occupation was encouraged both for physical exercise, as also to endeavour, if possible, to excite their mental faculties by making them keep the step when marching, and further to awaken attention, during various bodily manœuvres. Afterwards, the poor little fellows cheerfully sung a hymn, then performed some gymnastic exercises: and, notwithstanding the darkened state of their intellects, besides being confined in this small area, which constituted almost the sole outer world they knew, they appeared healthy, looked contented, and even happy, in spite of many mental, as likewise material, privations.

Another commendable feature should likewise be here mentioned—namely, that music, both vocal and instrumental, is much cultivated in this abode of affliction. The reverend almoner zealously promotes such sources of gratification, in which he is greatly aided by the assistant physician; besides various *frères* belonging to the establishment, who are often performers. These musical réunions are, however, encouraged more as recreations than like any scholastic instruction. A worthy *frère* plays on the piano, another on a bass fiddle, the clarionet, and so forth, whilst others, and patients, join in chorus. The evening previous to my visit, one of these much-appreciated musical parties had assembled, whereof ample evidence appeared in the large hall, into which I was shown next morning, prior to visiting the various dormitories; since various musical instruments, and other appurtenances used, during the fête, were still remaining in that apartment.

During the past year, sixty-two new patients were admitted, nineteen discharged cured, and twenty-eight died: which results show that, the proportion of deaths was even more numerous than actual recoveries. Amongst the cases terminating fatally, fifteen were reported as labouring under dementia, six had general paralysis, four were examples of mania, and the remaining three died from less defined varieties of mental disease. With regard to seasons, in reference to admissions, cures, and deaths, according to the experience of past years, it appears that, more patients were usually admitted during warm weather; as, for instance, in the months of May, June, and July. The largest number of cures being reported within six months after the patients' admission; whilst the deaths proved most numerous during the cold, or first months of each year. Again, respecting suicides, it may be mentioned as highly interesting that, from 1816 to 1852 inclusive, only eight cases of self-murder occurred among the whole male lunatics of this establishment. Such results may be partly explained by the fact that, great vigilance is constantly exercised, on the attendants' part, towards suspected suicidal patients, who are never left alone, but always associate with the other inmates. During day-time, that class of maniacs remain constantly under surveillance; and at night they sleep always in a dormitory surrounded by other lunatics, capable of watching over their conduct. Lastly, in the worst cases of that description, one religious brother belonging to this establishment occupies a bed adjoining the suspected individual, so as to notice every suspicious movement, and thus be ready for any emergency which may supervene.

The medical staff consists of one physician—the eminent M. Guislain—a consulting surgeon, and an assistant physician; but none of these officers reside on the premises. However, when the patients are removed to the new Asylum, a resident physician will be installed. The whole attendants are male persons; and consist of twenty-two religious brothers, four domestics, with four assistants: thus making, altogether, thirty individuals to superintend 260 lunatics—viz., one to every nine patients. Over these, a resident director presides, who is a clergyman, and takes the chief management. There is, besides, an almoner; the entire establishment being administered under the supreme direction of the City Hospital Commission. That body has constantly endeavoured, it is only just to observe on the present occasion, to do everything in their power to diminish the admitted insalubrity of this locality; and have, further, seldom been deterred from making any reasonable sacrifice to attain that result, or to promote the comfort of residents; whilst the zeal and talent of M. Guislain appear constantly exerted towards promoting other objects equally benevolent.

2. *Asylum for Females.*—The establishment which now comes under review is situated not far from the ancient Asylum for male patients just described. It lies in the same quarter of Ghent, being close to the street and canal already mentioned—having only intervening the large buildings, at present occupied as the College of Jesuits. According to an inscription still visible on a stone placed over the antiquated gateway, the year 1605 is stated to be the date of its foundation. The present structure was erected by the magistracy of Ghent, upon ground formerly constituting part of the ancient ramparts, but which now forms almost the centre of the modern city.

Being surrounded by streets, many private houses, besides public buildings, and having a large factory close to its very entrance—the noise of whose revolving machinery never ceases during day-time—the outward condition of this Asylum seems by no means favourable. In the interior, with reference to the actual number of its inmates, sufficient space appears wanting for the existing population. Hence, it is only through various ingenious combinations, carried forward by the constant zeal of managing authorities, that this institution has been made convenient, or able to contain comfortably its numerous residents under treatment. Like the establishment for males, it receives lunatics of the indigent classes belonging to Ghent; and likewise, by special permission, patients from other districts. The property belongs to the Civil Hospitals' Commission, and is managed under their administration. Although greatly superior to the male department in many attributes, nevertheless, impartial observers cannot but agree in the expressed opinion of several officials, that a time not distant must arrive, when some new locality will have to be chosen, and another structure erected, for the reception of indigent females; much of the same description as the building now in course of construction for pauper male lunatics. In the meantime, however, this institution continues to render important services to suffering humanity, being distinguished by the order, as also cleanliness everywhere prevalent, besides the care and attention exhibited towards patients. To carry out these important objects more effectually, the administration propose to add an adjoining house to the present accommodation; so that several further ameliorations may be accomplished, which cannot be now fully realized, in consequence of the limited space possessed, and from other existing inconveniences.

When perambulating the different dormitories, court-yards, and other appurtenances of this Asylum, although some appeared rather of a limited extent—owing to the nature of its ancient buildings, and confined interior precincts—the cleanliness, excellent ventilation, general tranquillity, and good order which prevailed throughout, were very gratifying to behold. Much attention appeared given to keep the various wards always thoroughly ventilated. This became the more necessary, although it was attended with greater difficulty in effecting, seeing apparatus had to be applied to an anciently constructed domicile like the present. M. Guislain has especially undertaken this very responsible task; and, judging from various effects already produced, by the machinery employed for that purpose, as likewise the absence of all unpleasant odours, when passing through different apartments at an early hour, visitors might conclude on such evidence that these hygienic operations have proved successful. Consequently, critics may fairly say that one step in advance had been made towards solving the much disputed problem—Can efficient ventilation be ever really accomplished?

Throughout, the wards looked very clean, the inmates tranquil, well clothed, and apparently contented. In one apartment I saw about 120 patients at work, many being then engaged in lace-making, which seemed to me of much better quality, if not finer, than that made by ordinary sane persons. Indeed, report states, the article manufactured in this establishment is highly esteemed, from its unusual cleanness and beautiful texture; these qualities being particularly noticed in a lace veil lately presented to H.R.H. the Duchess of Brabant. Subsequently, a large party were noticed at dinner, who then conducted themselves quietly, the same as ordinary persons, and really behaved very like rational creatures. In another apartment, upwards of a dozen young females—all idiots or imbeciles—were assembled at their singing-lesson, under the tuition of a zealous "sister." These poor girls sung delightfully, accompanied by their teacher on the piano, which made quite a musical treat; and as several juvenile performers were blind or dumb, while their execution hence seemed more surprising, this unexpected performance by intellectually bedimmed and unfortunate fellow-creatures caused us greater gratification. Many inmates seemed helpless from physical infirmities; but, considering their previous position in the external world, they now lived comparatively more comfortable.

The number of resident lunatics under treatment, on the day of my visit, amounted to 269 altogether, of whom 201 were considered incurables, and twenty-five as doubtful, in reference to any prospect of ultimate recovery; the remainder being classed as curable or recent cases. The agitated patients were reported at fifty; the epileptics comprised forty-seven examples; whilst the dirty furnished thirty instances. No person was under restraint of any kind whatever, nor in seclusion. Indeed, it may be added that, physical coercion in any form is very seldom employed at this establishment; the great objects constantly kept in view being to amuse and occupy the inmates, whereby tranquillity becomes promoted, at the same time that such means tend to improve their mental condition.

About half the entire population are usually engaged in some kind of employment. Many zealously spend hours in lace-making—the common occupation of females in this part of Flanders. Numbers work as mantua-makers; others in the laundry, and at wool-picking; besides a large proportion who attend to household and domestic duties; as, also, knitting stockings, or in making and mending clothes; of which the amount annually accomplished is considerable. It must however be added, that M.

Guislain does not consider the quantity of work done as always an unerring criterion of its utility. He even objects to any excessive development of physical labour in confined apartments, or close workshops, as thus imparting to the establishment an aspect of being a factory, a prison, or like ordinary depôts of mendicity. Further, M. Guislain thinks, unless the occupation chosen is carried out with discernment and caution, it may aggravate a lunatic's malady; whilst bodily labour which is severe, fatiguing, or too long continued, may do much harm; nay, even render the mental disease incurable.

During the past year seventy-seven new patients were admitted, and sixteen discharged cured, the deaths reported being thirty-two; thereby showing that recoveries were few, and fatal cases numerous. Amongst the latter, nine were cases of dementia, seven melancholia, and four general paralysis; the rest being mania and other varieties. Viewed with reference to the chief pathological phenomena observed, chest diseases were most numerous, affections of the abdominal viscera followed next, whilst the cerebral and nervous system supplied the fewest fatal illustrations.

Somewhat analogous to the experience observed amongst male patients, in reference to particular causes producing insanity, it may be also said that, moral influences were frequently reported, of which anxiety, chagrin, family misfortunes, devotion, and religious exaltation, seemed the most common; whereas the abuse of intoxicating liquors was very rarely observed. On the other hand, affections of the sexual organs, and disordered catamenia, not unfrequently appeared to have been a marked exciting cause of mental disease amongst female inmates.

Similar to the asylum for males, the medical staff at this institution consists of one attending physician, M. Guislain—its presiding genius—one consulting surgeon, and Dr. Vermeulen, the assistant physician; all being non-resident. Besides the "Sœur Supérieure" there are also thirty-one Sisters of Charity; of whom one is secretary, another music-mistress, while others are teachers of various departments, and chief superintendents; as also in other capacities, throughout different wards. To these, ten lay-female servants, with seven assistants, must be added; thus making altogether forty-eight actual attendants for 269 patients, or one to every six lunatic inmates.

Irrespective of the ordinary officials now enumerated, usually three male domestics belong to this establishment, who act as porters, messengers, and in out-door employments. Such appendages become absolutely necessary, when readers are informed, *No "sœur religieuse" attached to the institution ever goes beyond its threshold;* that being contrary to her sacred vows.

To these estimable females external society is closed for ever. Night and day must be wholly spent in assisting afflicted fellow-creatures. They sleep constantly on straw, and are devoid of all toilet luxuries; frequently fast, and pass much time in prayer, both late and early, at all seasons; yea, even when others are sound asleep. In truth, their whole existence seems a life of devotion and virtual self-sacrifice, which they here dedicate entirely to alleviate the sufferings of those insane persons who have come under surveillance. With reference to such sisters, one important feature should be further stated—viz., all rise regularly at 3·30 A.M., notwithstanding they were previously out of bed to assist at early religious duties in chapel, and although perhaps called up during night-time, to visit patients on emergencies. Many of the above enthusiastically unselfish ladies are persons of family, who have retired from the outer world with its varied allurements, so as to employ their mental energies and physical strength in attending upon the sick and unfortunate, without any prospect of fee or reward, on this side the grave. Nay, "sœurs" of the class described will frequently undergo privations, in order to assist others when required; and some will also contentedly injure present health, or peril life, in the great cause of benevolence, while aiding frail humanity.

Adjoining this establishment, but quite distinct in respect of all domestic arrangements, yet still under the same board of management, a "Maison de Santé" is attached, which has an entrance in the adjacent "Rue d'Assaut." This female precinct is exclusively appropriated for the reception of private insane patients, who pay from 600 to 3000 francs annually. The house in which they lodge is a large, commodious, and well-furnished mansion, having in front one small, although rather pretty garden. When I visited this department the number of inmates amounted to sixty lunatics, with fourteen Sisters of Charity, seven female servants, and two assistants. Thus making, altogether, eighty-two persons living within its enclosure; and hence, giving the proportion of one sane to nearly every three insane residents. M. Guislain is superintending physician, while the same assistant also officiates who is attached to the asylum for indigents.

3. *The Strop "Maison de Santé."*—Another establishment must likewise be noticed, although briefly, in connexion with the institutions for insane patients, located in or near Ghent, and to which M. Guislain is the attending physician, besides being further under the same directing management as the preceding. This asylum occupies an elevation not far from one of the city gates, is well ventilated, and has been constructed in an apparently salubrious locality. When I visited the institution, various buildings were in course of construction, including a new kitchen, and dormitory for dirty patients, in order thereby to afford additional accommodation. There is also a rather pretty garden attached. Still, to my apprehension, the whole enclosure then seemed too limited in extent for its present large population. Fifty patients were under treatment, all being of the upper and middle classes of society. The payments for board vary from 600 to 3000 francs annually; but sometimes beyond that sum, and even up to 6000 is paid, when any inmate requires a "frère" as his exclusive attendant, with also one or two apartments.

The head authority, or chief manager of this establishment is a clergyman, designated "*Le père supérieur.*" Besides having several lay-servants, for menial occupations, he has also under his direction twenty "*frères religieux,*" attached to an order whose denomination has escaped my remembrance. These officials overlook the various departments, and perform different assigned duties. In fact, they constitute the only attendants upon the patients ; no female being ever permitted to remain within the forbidden precincts—to them—of this "*sanctum insanorum.*" Moreover, unless in reference to medical treatment, with its chief direction, the whole internal management and discipline of this institution remains specially subjected to clerical superintendence.

During the past year, sixteen new patients were admitted, and seven discharged cured, while six deaths were reported. Hence, here as elsewhere, the ratio of recoveries proved small, and that of deaths large; when their several proportions are calculated according to the number of admissions.

4. *The New Asylum.*—Before taking leave of Ghent, and its insane establishments, some brief remarks respecting the large public asylum now constructing for indigent male lunatics, will neither seem out of place nor uninteresting. The locality chosen is situated at a short distance beyond the Bruges-gate, and occupies an agreeable, open position, not overlooked by any other buildings ; while otherwise it appears well selected for the purpose proposed. The structure is palatial-looking, has the form of a horse-shoe, rises two stories high, with various collateral appendages; and lastly, an elegant chapel will occupy its centre. The original plan and general programme was traced by M. Guislain ; who, besides being an eminent physician and zealous cultivator of science, is likewise an excellent practical engineer and architect. The execution and final completion of the entire building has been entrusted to M. Pauli, well known in Belgium as a man of talent ; and may be, it is confidently expected, ready for the reception of patients about eighteen months hence, or early in 1858 ; when there will exist altogether accommodation for 300 insane residents.

The erection of this public institution virtually constitutes a new era in Belgium, with reference to the management of lunatics; being the first receptacle of the kind in this country expressly constructed for their reception. It is likewise an eloquent manifestation of the great progress which has recently taken place in public opinion, respecting the objects to be kept constantly in view, wherever insane persons are brought together, for the purpose of treatment and protection. The new building, when finished, must further serve as a model for other establishments of the same description, which cannot fail to be constructed, before any long period elapses, in various Belgian provinces, now wholly devoid of such accommodation. Lastly, it is likely to become one of the most remarkable institutions throughout Europe, appropriated solely as an asylum for the insane.

The hospital administration of Ghent and general Government have both contributed towards the expenses incurred, which must amount to nearly a million of francs, before the whole structure is finished. But the money will be well expended, although hypercritics may likely say that too much has been laid out on its external embellishments, ornamented turret-looking chimneys, and minutely indented cornices. Such parties ought, however, to recollect that, being the first public asylum erected under government sanction, if it attracts more attention and discussion, even upon similar points, so much the better. Nay, should detractors object " to place paupers in palaces," which might be most justly said, occurs in the present instance, other provinces can avoid committing a similar error, in regard to future analogous establishments, by attending chiefly to internal arrangements.

Judging from the portion already finished, the entire construction will certainly prove very fine and imposing. The dormitories are not too large, or intended to contain so many inmates, as numerous similar apartments often seen on the Continent. They are lofty, spacious, and properly ventilated; having windows of greater magnitude than ordinary. Nowhere, unless at the new asylum of Auxerre, in France, have the sleeping rooms pleased me so much as those I noticed at this institution. Indeed, altogether, they appeared of a very superior description. Amongst many excellences which characterize its general features, in my opinion, the court intended for agitated patients is an exception, from not seeming well adapted for the purpose proposed. Being placed outside the round portion of this horseshoe-figured building, it wilt thus be more difficult to exercise constant surveillance over numerous excited inmates, than in square enclosures. A greater number of attendants will hence be required ; while one, at least, must always station himself near the central concave part of the outer encircling wall, in order to overlook, at the same time, as large a portion as possible of this really extensive enclosure. Still, that arrangement cannot remove the above objection; consequently, either more assistants will become necessary, or the space now intended for one must ultimately form two divisions.

Irrespective, however, of so very minor a fault in detail, and, perhaps, some other objections equally unimportant, there is yet no question regarding the undoubted superiority of this new construction, in every essential feature, over all previous asylums, appropriated for receiving lunatic patients, throughout Belgium. It cannot otherwise prove than of inestimable value to the afflicted insane poor of that country, and reflects great credit on the Government who promoted, as also those provinces contributing towards raising such an elegant structure. But to no person whatever will honour be more deservedly due than to M. Guislain—the original projector, who continues most zealous in his endeavours to ensure its perfect and final completion. Hence, the edifice should be named "L'Asile Guislain."

(To be continued.)

---

